# Raman characterization of *Avocado Sunblotch* viroid and its response to external perturbations and self-cleavage

**DOI:** 10.1186/2046-1682-7-2

**Published:** 2014-03-21

**Authors:** Gaston Hui-Bon-Hoa, Hussein Kaddour, Jacques Vergne, Sergei G Kruglik, Marie-Christine Maurel

**Affiliations:** 1 Unité 779, INSERM, 78 rue du Général Leclerc, 94276 Le Kremlin Bicêtre, France; 2 UMR 7205, Sorbonne Universités, UPMC Univ Paris 6, 4 place Jussieu, F-75005 Paris, France; 3 Laboratoire Jean Perrin (UMR 8237), Sorbonne Universités, UPMC Univ Paris 6, 4 place Jussieu, F-75005 Paris, France; 4 Laboratoire Jean Perrin (UMR 8237), CNRS, 4 place Jussieu, F-75005 Paris, France

**Keywords:** Viroid, RNA conformation, Self-cleavage activity, D_2_O perturbation, Temperature unfolding, Raman spectroscopy

## Abstract

**Background:**

Viroids are the smallest pathogens of plants. To date the structural and conformational details of the cleavage of *Avocado sunblotch* viroid (ASBVd) and the catalytic role of Mg^2+^ ions in efficient self-cleavage are of crucial interest.

**Results:**

We report the first Raman characterization of the structure and activity of ASBVd, for plus and minus viroid strands. Both strands exhibit a typical A-type RNA conformation with an ordered double-helical content and a C3′-endo/anti sugar pucker configuration, although small but specific differences are found in the sugar puckering and base-stacking regions. The ASBVd(-) is shown to self-cleave 3.5 times more actively than ASBVd(+). Deuteration and temperature increase perturb differently the double-helical content and the phosphodiester conformation, as revealed by corresponding characteristic Raman spectral changes. Our data suggest that the structure rigidity and stability are higher and the D_2_O accessibility to H-bonding network is lower for ASBVd(+) than for ASBVd(-). Remarkably, the Mg^2+^-activated self-cleavage of the viroid does not induce any significant alterations of the secondary viroid structure, as evidenced from the absence of intensity changes of Raman marker bands that, however exhibit small but noticeable frequency downshifts suggesting several minor changes in phosphodioxy, internal loops and hairpins of the cleaved viroids.

**Conclusions:**

Our results demonstrate the sensitivity of Raman spectroscopy in monitoring structural and conformational changes of the viroid and constitute the basis for further studies of its interactions with therapeutic agents and cell membranes.

## Background

Viroids are the smallest pathogens of plants; they are characterized by a compact rod-like circular RNA 246–475 nucleotides long [1]; they have no envelope, no capsid and they do not code for any protein. Viroids are divided into two families, the *Avsunviroidae* such as *Avocado sunblotch* viroid (ASBVd) that all possess a catalytic RNA with a hammerhead ribozyme (HHR) motif responsible for a crucial cleavage step during viroid replication and the *Pospiviroidae*. The 3D structure of an HHR [2,3] is composed of three helical junctions (I, II, III) with a core of invariant nucleotides required for its activity. The HHR motif of about 35 nucleotides contains a well-conserved single strand region including mandatory nucleotides required for efficient catalysis. Metal ions are involved in HHR activity within the cleavage sites (C–U and C–G). Studies of *Chrysanthemum chlorotic mottle* viroid (CChMVd) showed that RNA sequences peripheral to the ribozyme can enhance self-cleavage activity [4,5]. Cleavage of HHR is a transesterification reaction that converts a 5′, 3′ diester to a 2′, 3′ cyclic phosphate diester via an SN2 mechanism [6]. During replication, (+) and (-) complementary strand sequences of *Avsunviroidae* are generated through the symmetric rolling circle mechanism [7,8]. The analysis of the ASBVd contents in avocado extracts [9] revealed the presence of RNA of both polarities in multimeric forms, from monomers to octamers for ASBVd(+) and monomers to dimers for ASBVd(-). This difference in oligomeric sizes reveals a less efficient *in vivo* cleavage activity of ASBVd(+) than of ASBVd(-) than was observed by *in vitro* cleavage. The viroid moves within the cell thanks to intrinsic RNA signals but it is also likely that it recruits supporting protein or RNA factors. It has been shown that complexes between viroids and specific tRNAs exist under physiological conditions. The fact that the concentrations of tRNAs and viroids may be sufficiently high in the cell, suggests that these complexes may also be formed in vivo with functional relevance [10]. Because of the diversity of structures and dynamics that participate in viroid trafficking within the cell and between cells, as well as during infectivity, it is of crucial interest to characterize the structural elements involved in viroid processing. The predicted structures of ASBV(-) and (+) strands have been studied experimentally by Navarro and Flores [11,12]. These structural components might represent the driving force necessary for the viroid to penetrate the cell as well as to interact with cell components. Furthermore, structural elements possibly correspond to functional changes during the life cycle of the viroid [11]. On the other hand, the reconstructed sequence of proto-tRNA^Gly^ was found to have a sequence capable of adopting a hammerhead structure. Additionally, from base-sequence alignments it is suggested that proto-tRNA^Gly^ was possibly a viroid-like self-cleavable ribozyme with a hammerhead-like motif [13].

Despite the large amount of information regarding the molecular biology of *Avsunviroidae*[8], to date little is known regarding the structure and conformational aspects of the cleavage of minus and plus ASBVd strands and the catalytic role of Mg^2+^ in efficient self-cleavage of such viroids [12,14]. Raman spectroscopy has a great potential as a sensitive probe of molecular structure, not only of highly concentrated fibrous forms but also of dilute nucleic acid solutions under near-physiological conditions [15]. Vibrational spectra contain a great deal of information about the molecular structure and dynamics of RNA [16,17]. The common goal in such investigations is to establish a reliable correlation between vibrational spectra and specific structural features of RNAs and their biologically important complexes. In particular Raman vibrational modes of the RNA phosphodiester group and base rings are sensitive to the conformation of the RNA backbone [18,19]. Libraries of empirically established correlations between Raman spectra and biologically important active nucleotide structures in the A, B and Z forms have been compiled [19-24], although some correlations involving Raman conformation markers of RNA structures are as yet not fully interpretable and are being intensively investigated. It was found that Raman spectra of different types of RNA are rather similar and possess about 30 characteristic lines that arise from the vibrations of the sugar-phosphate backbone of the RNA and bases [25-27]. Bands assignment in Raman spectra of tRNAs for example, is based on the comparison with the spectra of homopolymers of the four regular ribonucleotides (poly (G), poly (C), poly (A) and poly (U)) [23,28-30].

Here we report the first structural characterization of the minus and plus polarities of ASBVd, by Raman spectroscopy with near-infrared laser excitation light at 780 nm. To obtain further information about the dynamics and stability of such viroids, deuteration (D_2_O/H_2_O exchange) and thermal unfolding techniques were used, in conjunction with gel electrophoresis study.

## Results and discussion

### Raman bands assignment and structural parameters for ASBVd

**Figure 1:**
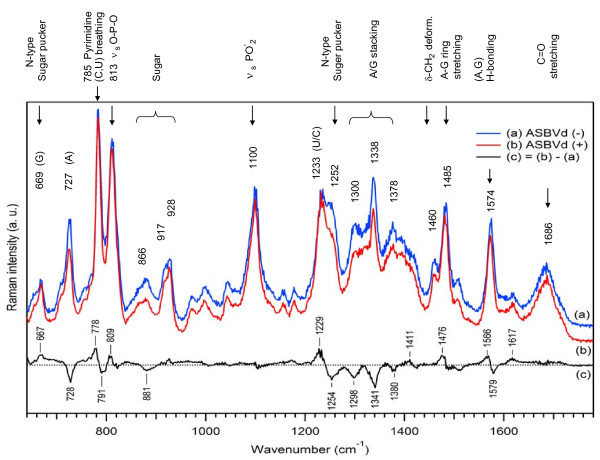
**Assignment of major Raman bands of ASBVds.** Raman spectra of ASBVd(-) ((a), blue curve) and ASBVd(+) ((b), red curve) are obtained in aqueous cacodylate buffer, in the absence of Mg^2+^. Both spectra were normalized using the Raman band at 1100 cm^-1^ taken as an internal intensity standard, and the contributions from the cacodylate buffer and quartz cell were subtracted. The black curve (c) is the difference Raman spectrum obtained by (1:1) subtraction of the spectrum (a) from spectrum (b). The difference spectrum (c) was multiplied by a factor of 3 to visually enhance the resulting spectral changes.

Figure 1 presents the Raman spectra of the minus (curve (a)) and plus (curve (b)) strands of ASBVd, in the range 640–1780 cm^-1^, in aqueous cacodylate buffer, at 20°C. These spectra consist of many well-resolved lines that can be grouped into five distinct regions.

(I) Low-frequency region between 600 and 850 cm^-1^ corresponds to vibrations of the nucleotide heterocyclic rings as well as of the phosphodiester backbone. The weak line at 669 cm^-1^ and the stronger line at 727 cm^-1^ correspond to breathing vibration of the (G) and (A) purine bases respectively. The characteristic feature in this region is the existence of two strong sharp bands, at ~785 cm^-1^ and ~813 cm^-1^. The band at ~785 cm^-1^ is assigned to ring breathing of pyrimidine (C,U) bases [17,24], and the band at ~813 cm^-1^ is attributed to a symmetric stretching vibration ν_s(C–O-P-O-C)_ of the phosphodiester linkage of the A-form RNA. Both the intensity and frequency of this latter band are sensitive to bond angle deformations of the RNA backbone [17,26,31,32]. In B-DNA, the strong line at 813 cm^-1^ is transformed into a weak broad shoulder around 835 cm^-1^, both bands being independent on base composition [33]. Protonation of poly(rA) at pH 5 results in high-frequency shift 813 → 824 cm^-1^ while the phosphodioxy (PO_2_^-^) band at 1100 cm^-1^ (see below) is not affected [34]. It is known that ordered polyribonucleotides exhibit a strong Raman line at 813 cm^-1^, while no line at this frequency is observed in their disordered structures upon thermal denaturation [30]. As the intensities of the bands at 785 cm^-1^ and 813 cm^-1^ vary relatively to each other at different RNA configurations, we have defined the ratio r_conf_ = I_785_/I_813_ to characterize the degree of A-type phosphodiester conformation of the RNA. The spectral overlap between these bands renders the accurate measurement of their intensities difficult; to overcome this problem, we have adopted the method based on peak heights for the estimation of r_conf_.

(II) Weaker bands in the region 850–1050 cm^-1^ originating predominantly from sugars are also sensitive to backbone geometry and secondary structure.

(III) In the range 1050–1150 cm^-1^, a strong Raman band appears at ~1100 cm^-1^. This band is assigned to the symmetric stretching vibration ν_s_(PO_2_^-^) of the phosphodioxy group, sensitive to changes in the electrostatic environment of the phosphate group [35]. At the same time, the PO_2_^-^ groups essentially constitute an independent oscillator, insofar as they do not couple extensively to the motions of the C_3_’ and C_5_’ ribose atoms, being largely insensitive to the geometry of sugar-phosphate diester linkages. Therefore, at constant ionic strength, this phosphodioxy stretching vibration is expected to have about the same Raman intensity for all polynucleotides and RNA adopting the same helical structure [23,36]. Its intensity depends only on the number of anionic oxygen atoms and serves as a useful internal marker [32], thus normalization on the intensity of this band allows direct comparison between Raman spectra of various nucleic acids. Since the magnitude I_813_ is directly proportional to the number of phosphodiester linkages in ordered configurations and that of I_1100_ is independent of such structural factors, we have defined the ratio r_2_ = I_813_/I_1100_ to characterize the amount of the secondary structure (double helical content) [17,28,32]. The precision of the determination of the ratio r_2_ is about ±0.05.

(IV) The frequency region 1150–1600 cm^-1^ contains Raman bands of purine and pyrimidine coupled nucleotide vibrations that are sensitive to ring electronic structures such as base stacking rearrangements, ligation with metal binding, etc. For example, ν_(pyr + imidazole)_ at 1300 cm^-1^ and 1378 cm^-1^ are composite vibrations of adenine or guanine ring systems consisting of fused cycles of six-membered pyrimidine and five-membered imidazole (Im) rings, while ν_(Im)_ at 1338 cm^-1^ arises from imidazole ring vibration alone [37]. In our subsequent analysis, the Raman normalized intensity ratio r_stack_ = (I_1300_ + I_1378_)/I_1338_ was used to characterize base-base stacking interactions. Indeed, in the study of short oligo- and polynucleotides, the value of this parameter has been shown to decrease linearly with the logarithm of the concentration of all three (adenine, AMP and ATP) bases [38], so that Raman band intensities decrease (hypochromicity) when base-base stacking interactions occur. Strong Raman bands around 1233 cm^-1^ are assigned to U/C ring stretching vibrations which change strongly in intensity upon thermal unfolding (hyperchomicity). Strong Raman band at 1252 cm^-1^ is assigned to the C_3_’-endo/anti (or so-called N-type) sugar pucker conformation. The ribose in the RNA is manifested by a Raman band at about 1460 cm^-1^ attributed to methylene twisting ν_t(δ–CH2)._ Other strong bands, at 1485 cm^-1^ and 1574 cm^-1^, are attributed to purine A-G cycle vibrations along the long/short axis and hydrogen bonding of bases (A,G) [39].

(V) The H-bonding region is located at 1600–1760 cm^-1^ and shows a broad band centered near 1640–1686 cm^-1^ which is attributed to the carbonyl C = O stretching modes of pyrimidines. The intensity and position of this composite band is sensitive to its coupling with the N-H deformation mode of the bases and to thermal denaturation or D_2_O perturbation, reflecting alteration of hydrogen bonding states between the exocyclic donor and acceptor groups of the bases [31].

The Raman spectrum of ASBVd(+) (Figure 1(b)) is rather similar to that of ASBVd(-) (Figure 1(a)), except for some small but detectable differences in (i) the pyrimidine base stretching vibration (at 785 cm^-1^ and 1233 cm^-1^) and purine rings stretching (727 cm^-1^ and 1485 cm^-1^), (ii) the sugar-phosphate backbone regions (at 813 cm^-1^ and 866 cm^-1^), (iii) the base-stacking (at 1300–1380 cm^-1^) and the H-bonding in the double bond region (at 1540–1600 cm^-1^) but not in the phosphodioxy intensity and position. Figure 1(c) presents Raman difference spectrum (multiplied by a factor of 3) obtained by subtracting the spectrum of ASBVd(-) from that of ASBVd(+).

### Structure and activity of ASBVd(-) and ASBVd(+)

#### Double helical content

From the data of Figure 1(a) we have calculated that the r_2_ parameter for the ASBVd(-) viroid is equal to 1.23, while it is equal to 1.25 in formylmethionyl-tRNA [25] and 1.33 in the ribozyme D5-PL-RNA [12]. For each of the ordered polyribonucleotides listed in Table IIA of Thomas and Hartman [40], the value of r_2_ was found to be constant at 1.64, indicating the conformational similarity of (-C–O-P-O–C–) linkages. In addition the ratio r_2_ approaches zero in completely disordered structures [36,41]. Hence, the ratio r_2_ observed for ASBVd(-) and model compounds can be converted into the percentage of ordered polyribonucleotide RNAs using the factor 1.64 as a coefficient of division. The estimate of the number of phosphodiester groups in the ordered region of the viroid RNA, obtained using this procedure, is ~75% whereas it is 76% for formylmethionyl-tRNA and 81% for the ribozyme D5-PL-RNA [17]. Subtle conformational differences in the structure of the RNA in ASBVd(-), as compared to the ribozyme D5-PL-RNA are interpreted by the existence of a mixture of serial arrangements of double-helical sections and internal loops.

The double helical content in ASBVd(+) (Figure 1(b)) is equal to r_2_ = 1.28, leading to the estimation of ~78% of the RNA nucleosides in ordered configurations. Thus the secondary structure of the RNA in ASBVd(+) is more structured than that in ASBVd(-). In addition, the secondary structure differs slightly in the geometry of the purine bases that exhibit a moderate decrease in intensity (about 15%) at 727 cm^-1^. Such a difference is presumably due to ring vibrational coupling between purine bases and the riboses. Our results on double helical content are to be compared to those of Thomas et al. [41] that give the values of 95%, 85%, 87% for 16S rRNA, 23S rRNA and R17 RNA respectively. The secondary structures of these RNAs are more ordered and structured than in the case of ASBVd viroid. It is worth noting that the value of r_2_ as determined from Raman intensity measurements is originated in RNA backbone configuration and not in the bases. So it does not depend on the base sequence as well as on G:C content [41].

### Phosphodiester conformations

The conformation of the sugar-phosphodiester group of ASBVd(-) is characterized by the parameter r_conf_ = 1.14. Interestingly, for a canonical A-form RNA, the Raman doublet 785/813 cm^-1^ exhibits typically a higher Raman intensity of the 811–814 cm^-1^ band as compared to that at 785 cm^-1^ leading to r_conf_ <1. At the other extreme, for soluble DNA in a “canonical” B conformation, the Raman band at ~813 cm^-1^ transforms into the broad shoulder at ~835 cm^-1^ leading to r_conf_ → ∞. However, in the presence of low salt and 75% humidity, the symmetric phosphodiester band at ~813 cm^-1^ appears in the DNA fiber yielding r_conf_ = 1 [17]. The same conformation was found for ribozyme D5-PL-RNA where r_conf_ = 1.01 [12]. The difference in the r_conf_ parameter between ASBVd(-) and fiber DNA indicates that although both nucleic acids are in the A conformation, they differ in subtle bond-stretching vibrations located in the –C–O-P-O–C– networks and possibly also in the ribose ring [17,32]. A big difference in r_conf_ was found in the structure of formylmethionyl-tRNA [25] where r_conf_ =1.6, although it exhibits similar to ASBVd ordered secondary structure characterized by the parameter r_2_. This result can be interpreted by a difference in the local phosphodiester conformation of the respective A-type structures.

The value r_conf_ for ASBVd(+) (Figure 1(b)) is the same as for ASBVd(-). At the same time a slight frequency downshift is observed in the region of the –C–O-P-O-C– vibrational mode at 813 cm^-1^ (Figure 1(c)), indicating a slight difference in the rigidity of the phosphodiester backbone of ASBVd(+). On the contrary, the phosphodioxy stretching mode at 1100 cm^-1^ in both minus and plus strands of the viroid remains the same, both in intensity and frequency.

### Base-pairing and base-stacking

Normalization of viroid Raman spectra on the intensity of ν_s_(PO_2_^-^) at 1100 cm^-1^ permits the characterization of the strength of each base-specific vibrational modes and also of the interactions of various nucleic acids with the medium. In particular, for free ASBVd(-) in the absence of Mg^2**+**^, Figure 1(a) shows that the breathing vibration of adenine produces a rather strong band at 727 cm^-1^ with a normalized intensity of I_727_ = 0.7, while the breathing vibration of guanine at 669 cm^-1^ exhibits much weaker intensity (I_669_ = 0.3). The Raman bands resulting from (A-G) ring stretching vibrations at 1485 cm^-1^ and the H-bonding at 1574 cm^-1^ are strong markers with increased intensities of 0.9 and 0.7 respectively. The H-bonding in the carbonyl C = O stretching region manifests itself in a broad band with a maximum around 1686 cm^-1^ and with an intensity of 0.5. In the case of ASBVd(+) (Figure 1(b)), there are no appreciable changes in the carbonyl stretching region as compared to ASBVd(-), while there is a slight frequency downshift at 1574 cm^-1^, which is the H-bonding marker between A and G bases. On the other hand, the base-stacking mode which contributes importantly to the conformation and stability of the RNA structure is of major interest. The previously defined parameter r_stack_ shows that for free ASBVd(-) and ASBVd(+), it is equal to 1.35 and 1.42 respectively thus indicating a slight decrease in base-base interactions in the ASBVd(+) structure and emphasizing small but noticeable differences in the conformation of the plus versus the minus strand.

### Sugar puckering coupled to (G, U) bases

As indicated in the previous section, the Raman frequency of the purine (G) base for ASBVd(-) is located at 669 cm^-1^ with a weaker intensity than the Raman band of the (A) base located at 727 cm^-1^. However the frequency and intensity of these bands depend on the local conformation of the nucleotides [42]. Because the glycosidic bond (N-C_1_’) is close to the base moiety, there is appreciable vibrational coupling between the weak stretching vibration of the guanine and the stronger one of the ribose. Thus the parameters of the two Raman bands may vary depending on the geometry of the nucleotide bases (“syn” or “anti” overall rotation geometry with respect to the sugar) [43], and could provide information about the torsional rotations of the glycosyl bonds and their flexibility. In ASBVd(+), the stretching mode at 727 cm^-1^ for purine bases exhibits a moderate decrease in intensity (about 15%) as compared to ASBVd(-) (Figure 1(c)). This can be interpreted as a difference in the ring vibrational coupling between purine bases and ribose in ASBVd(+), leading to a small local difference between two viroids in the sugar puckering conformation, and suggesting the existence of a more compact and rigid geometry in the backbone of ASBVd(+).

Interestingly, the conformational features of the nucleotides involved in RNAs motifs are determined by the sugar pucker structures and the bases rotation geometries. For instance, the hairpins contain conformations of two types, the so-called “N-type” and “S-type” conformations. The “N-type” structure is characterized by Raman marker band around 1252–1254 cm^-1^ which is assignable to C–3′ atom of the sugar in “endo” position and the corresponding base in “anti” rotation geometry. The “S-type” conformation exhibits Raman marker band around 1267 cm^-1^, which is assignable to the C–2′ atom of the sugar in “endo” position and the base in “anti” rotation geometry. Another Raman marker band located in the low frequency region of guanine nucleoside stretching around 669 cm^-1^ is assignable to C_3_’-endo/anti conformation (“N-type”), while the C_3_’-endo/syn orientation of any G base with respect to its adjacent sugar is characterized by Raman marker located at 1320 cm^-1^[43]. In our case the Raman spectrum of free ASBVd(-) in Figure 1(a) shows the presence of a strong band at 1252 cm^-1^ (I_1252_ = 0.84) and a weaker band at 669 cm^-1^(I_669_ = 0.3), both bands being assignable to nucleosides in C_3_’-endo/anti conformations. The same vibrational stretching bands are also observed in ASBVd(+) (Figure 1(b)) with only slightly different intensities (I^+^_1252_ = 0.79 and I^+^_669_ = 0.39). This result suggests that in both minus and plus strands of ASBVd, all sugars adopt the N-type conformation of the sugar puckers. Indeed, for both types of ASBVd, neither Raman marker at 1267 cm^-1^ nor at 1320 cm^-1^ were observed in our study.

### *In vitro* activity

**Figure 2:**
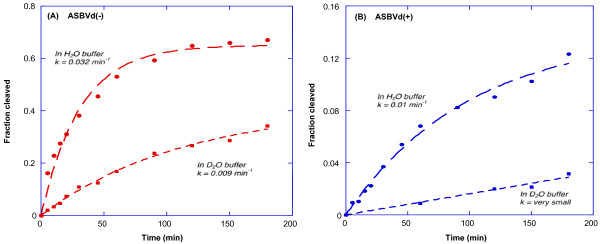
**Kinetics of the self-cleavage activity of ASBVds.** The kinetic curves of ASBVd minus (panel **A**) and plus (panel **B**) strands are obtained in cacodylate buffer in H_2_O (circles, long dashes) and in D_2_O (squares, short dashes).

Controls of the self-cleavage activity of ASBVd(-) and (+) have been performed by gel electrophoresis at 45°C, in two different solvent conditions, H_2_O and D_2_O. The cleaved fraction was plotted (Figure 2) versus time and fitted with a single exponential growth equation. Figure 2A reveals that ASBVd(-) is 3.5 times more active in cleaving in H_2_O than in D_2_O. The rate constants are respectively 0.032 and 0.009 mn^-1^. After 200 minutes, the cleaved fraction was ~65% in H_2_O and ~32% in D_2_O. The comparison of the activity profiles (Figure 2A versus Figure 2B) shows that ASBVd(-) is about 3.5 times more active than ASBVd(+) in H_2_O where the rate is 0.01 mn^-1^. Thus even in the presence of D_2_O, ASBVd(-) is as active as ASBVd(+) in H_2_O. Besides, the activity of ASBVd(+) in D_2_O is almost completely quenched (Figure 2B). Such a difference in self-cleavage activity corroborates well with the differences in structural dynamics between ASBVd(-) and ASBVd(+) as found from Raman spectra in D_2_O (see below).

Isotope substitution (H_2_O → D_2_O) is usually utilized for elucidation of the mechanism of enzymatic reactions [15]. As an example, Takagi *et al.* investigated the kinetics of hammerhead ribosyme reaction for H_2_O and D_2_O solvents, in the presence of high concentration of NH_4_^+^ and Li^+^ ions [44]. They obtained the higher values of the ratio k^H2O^_app_/k^D20^_app_ in the former case and concluded that proton transfer occurs only in the NH_4_^+^-mediated reaction and not in the Li^+^-mediated one.

In our case of ASBVd(-) viroid in the presence of 20 mM Mg^2+^ ions (Figure 2A), this ratio is equal to 3.5, suggesting a mechanism of acid/base catalysis involving proton transfer.

### Effect of physico-chemical perturbations on the viroid structure

#### Solvent deuteration and exchangeable protons

**Figure 3:**
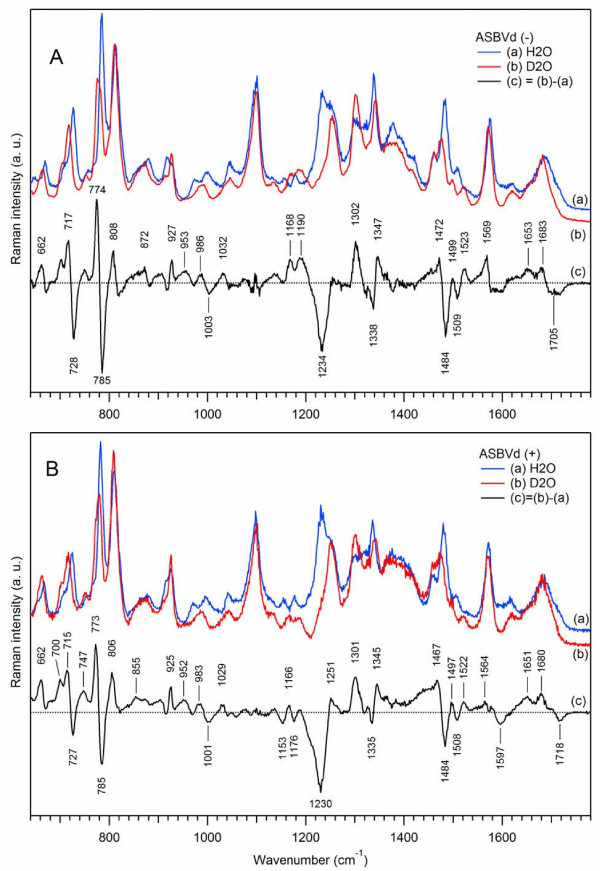
**Effect of deuteration on Raman spectra of ASBVds.** The Raman spectra of minus (panel **A**) and plus (panel **B**) strands of ASBVd are obtained in cacodylate buffer, in the absence of Mg^2+^. Blue curve (a) correspond to Raman spectra in H_2_O; red curve (b) correspond to Raman spectra in D_2_O. All spectra were normalized using the Raman band at 1100 cm^-1^; the contributions from the cacodylate buffer and quartz cell were subtracted. The difference spectrum (c) (c = b-a, black curve) correspond to spectral changes due to H to D atom substitution.

Raman vibrational spectroscopy of different isotopomers is a useful tool in studies of intermolecular interactions in liquids, as the perturbed spectra in the deuterated environment specifically characterize the mobile hydrogen atoms in the accessible sites of the viroid. In addition, it is currently applied to nucleic acids studies to perturb sensitive frequencies and to identify orientation of phosphodiester linkage and sugar pucker conformations as well as coupling of the double-bond stretching and N-H deformation vibrations of the bases [31,45]. Figure 3 shows that Raman spectra of deuterated viroid (-) and (+) strands (curves (b)) are substantially different from their non-deuterated analogs (curves (a)). The spectral changes occur in three major frequency regions. Let us first consider the case of ASBVd(-) (Figure 3A).

*In the 600–850 cm*^*-1*^*region*, the ring-stretching vibrations of purine and pyrimidine exhibit shifts to lower frequencies. The Raman stretching vibrations of adenine at 727 cm^-1^ and guanine at 669 cm^-1^ are downshifted to 717 cm^-1^ (Δν = -11 cm^-1^) and 662 cm^-1^ (Δν = - 7 cm^-1^) respectively, with moderate intensity changes. This is interpreted in terms of H to D atom substitution and the removal of the coupling between the ribose and the purine bases upon deuteration. There was thus more freedom for the base stretching vibrations and for the torsional rotations of the glycosyl bonds, leading to changes of the backbone conformation. Interestingly, in the presence of D_2_O, another A-type structure of the viroid is observed. The position and the shape of the doublet 785/813 cm^-1^ changes to 776/812 cm^-1^, with an important intensity decrease of the (C,U) pyrimidine ring vibrational peak at 776–785 cm^-1^ and a slight intensity increase of the phosphodiester symmetric peak at 811–813 cm^-1^. The r_conf_ parameter decreases by 25%, from r_conf_ = 1.2 in H_2_O to r_conf_ = 0.9 in D_2_O, and the overall amount of double-helical content is increased by 9% (r_2_ changes from 1.28 to 1.40) upon deuteration. Interestingly, the phosphodioxy Raman marker at ~1100 cm^-1^ is not perturbed.

*In the 1160–1580 cm*^*-1*^*region*, three main changes in the Raman markers are found: i) the strong line of the U/C ring stretching vibration located at 1233 cm^-1^ in water disappears in D_2_O while an intense Raman line at 1302 cm^-1^ of pyrimidine + imidazole vibration of the adenine ring appears in the spectrum of deuterated ASBVd(-), indicating that the external “in plane” C-N stretching vibrations of purine and pyrimidine rings are sensitive to D_2_O. ii) The stacking parameter r_stack_ which is equal to 1.66 in D_2_O as compared to 1.32 in H_2_O increases by about 34%. iii) The band at ~1485 cm^-1^ assignable to A/G purine ring stretching is also very sensitive to D_2_O perturbation giving a strong negative peak in the difference spectrum. These changes, concomitant with the change of the double helical content, clearly demonstrate that D_2_O perturbs some internal loops, base-base interactions and increases double helical rearrangement in the sugar-phosphate backbone leading to a new conformation and rigidity of the structure.

*The broad H-bonding Raman band in the carbonyl region (1640–1740 cm*^*-1*^*)* becomes more structured: the difference Raman spectrum (Figure 3A(c)) shows peaks at 1683 cm^-1^ and 1653 cm^-1^ with a shoulder at ~1621 cm^-1^. The appearance of these new lines presumably results from the removal of the coupling between the C = O double bond stretching and the N-H deformation vibrations of the bases upon deuteration. Since the N-D deformation vibrations occur at much lower frequencies around 1200 cm^-1^, the new spectral pattern in the high-frequency range results solely from the C = O stretching of the uracil base: an increase in the number of unpaired uracil residues (peak at 1653 cm^-1^) was observed as compared to the paired uracil residues (peak at 1683 cm^-1^).

In the case of ASBVd(+) (Figure 3B), the spectral changes caused by solvent deuteration are generally very similar to those for ASBVd(-). However, the intensity changes in Raman difference spectrum of ASBVd(+) (Figure 3B(c)) are weaker than for its minus-strand analogue (Figure 3A(c)), suggesting that the structure of ASBVd(+) is more rigid, containing less internal loops [11] and is less accessible to D_2_O. These results suggest that the exchangeable protons and H-bonding are located mainly in the phosphodiester backbone and in the (U, A) bases around 1234 and 1484 cm^-1^ respectively. The base-stacking is also perturbed, but to a lesser extent for ASBVd(+).

### Thermal unfolding

**Figure 4:**
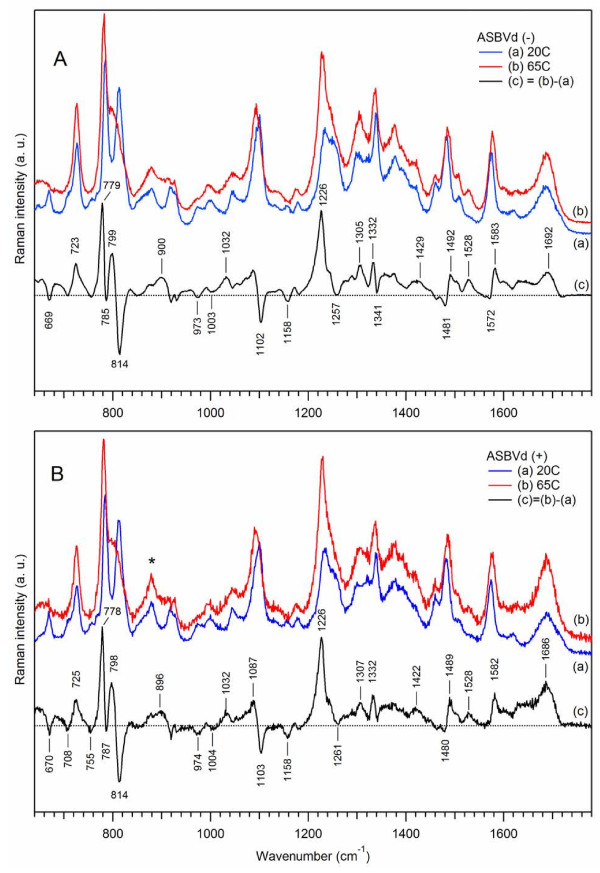
**Effect of temperature on Raman spectra of ASBVds.** The Raman spectra of minus (panel **A**) and plus (panel **B**) strands of ASBVd are obtained in cacodylate buffer, in the absence of Mg^2+^. Blue curve (a) correspond to Raman spectrum taken at 20°C; red curve (b) correspond to Raman spectrum taken at 65°C. All spectra were normalized using the Raman band at 1100 cm^-1^; the contributions from the cacodylate buffer and quartz cell were subtracted. The difference spectrum (c) (c = b-a, black curve) correspond to spectral changes due to temperature changes.

Application of an unfolding temperature to ASBVd reveals several characteristic spectral features. Figure 4 presents Raman spectra at 20°C (curves (a)) and at 65°C (curves (b)), together with the difference spectra (curves (c)) that were obtained by (1:1) subtraction of the low-temperature spectrum from the high-temperature one. Note that the intensity of the phosphodioxy stretching vibration around 1100 cm^-1^ was used as the reference for spectra normalization, prior to spectra subtraction.

As a general conclusion, both strands of ASBVd exhibit temperature-induced spectral changes quite characteristic for polyribonucleotides. The intensity changes of the Raman markers are very similar for ASBVd(-) and ASBVd(+). More specifically, at 65°C, there are pronounced changes in the position and intensities of several Raman markers. The strong symmetric (-C-O-P-O-C-) phosphodiester mode at 813 cm^-1^ decreases in intensity and is transformed into a broad shoulder at 779 cm^-1^. Frequency downshift is also observed for the pyrimidine stretching mode at 785 cm^-1^ which slightly increases in intensity. Other prominent spectral changes involve the frequency downshift of the phosphodioxy symmetric stretching (from 1103 cm^-1^ to 1087 cm^-1^) and the enhancement of the hyperchromic band around 1226 cm^-1^ corresponding mainly to a U stretching mode.

The ratio r_2_ decreases from 1.2 to 0.79, the ratio r_conf_ increases from 1.2 to 1.8, and, together with the 813 cm^-1^ band transformation, indicate the loss of the A-type structure of ASBVd which is characterized by ~48% ordered double helical content at 65°C as compared to ~75% at 20°C. Temperature unfolding considerably perturbs the conformational structure of the sugar-phosphodiester backbone for both types of viroid. The bands at 671 cm^-1^ and 918–928 cm^-1^ decrease in intensity suggesting some loss of nucleotide conformation and backbone geometry. A hyperchromism of the 1228 cm^-1^ band and an increase of the r_stack_ parameter from 1.36 to 1.45, both indicate the loss of base-stacking and destabilization of the double-helical structure. A moderate intensity increase in the carbonyl region around 1690 cm^-1^ reflects the rupture of hydrogen bonds between bases at 65°C.

We note that the effect of temperature is quite different from that of solvent deuteration (Figure 4 versus Figure 3).

### Mg^2+^ binding before self-cleavage

It is known that divalent metal cations influence the RNA structure and are potential regulators of ASBVd. Indeed, the N7 site of G is an important site for metal cation interactions which bind preferentially to GC rather than to AT regions [46]. Phosphodioxy groups could also be targets. However, the affinity of the interactions with phosphates and specific bases depends on the nature of metal cations (transition metal versus alkaline earth metal) as well as on the type of nucleic acid [44].

**Figure 5:**
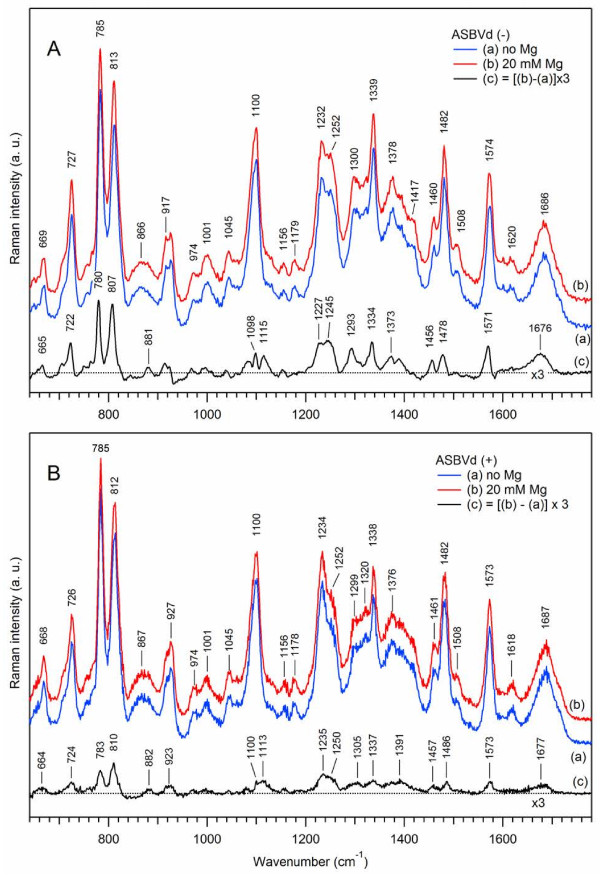
**Effect of Mg**^**2+ **^**binding on Raman spectra of ASBVds.** The Raman spectra of the minus (panel **A**) and plus (panel **B**) strands of ASBVd are obtained in cacodylate buffer. Blue curve (a) correspond to Raman spectrum in the absence of Mg^2+^; red curve (b) correspond to Raman spectrum in the presence of 20 mM Mg^2+^. Spectra were not normalized in order to see the effect of Mg^2+^ on the phosphodioxy band at 1100 cm^-1^, but the contributions from the cacodylate buffer and quartz cell were subtracted. The difference spectrum (c) (c = b-a, black curve) was multiplied by a factor of 3 to visually enhance the resulting spectral changes corresponding to Mg^2+^ binding at 20°C.

Figure 5 shows the effect of adding 20 mM Mg^2+^ on the structure of ASBVd, at 20°C. The resulting small but reproducible spectral changes reveal structural perturbations within specific heterocyclic bases. Purine (A) ring stretching vibrations at 727 cm^-1^ increase by ~7% in intensity, while there is no or a very small effect in the “N-type” sugar pucker Raman bands (~4%). A slight methylene δ–CH_2_ deformation change (at 1460 cm^-1^) with a small frequency downshift of ~4 cm^-1^ is observed. In addition, the intensity increase approaching 6-8% for 785 cm^-1^ and 813 cm^-1^ bands respectively, with no changes of the r_conf_ = 1.14 and r_stack_ = 1.4 parameters suggests that there is a small local change in the nucleotide geometry and in the 3′-endo ribose conformation in ASBVd(-), with no perturbation of the overall backbone geometry. Note that for ASBVd(+), the intensity increase is less pronounced, being ~5%. At the same time, the phosphodioxy symmetric stretching band at 1100 cm^-1^ is slightly perturbed in intensity and position (about 3%). This effect is presumably due to charge neutralization rather than Mg^2+^ cation binding to the phosphate. Finally, in the purine base stretching, H-bonding and C = O double bond regions, located around 1480–1700 cm^-1^, the intensities of the Raman bands also slightly increase. The results clearly favor a specific Mg^2+^ cation binding to the heterocyclic bases of the ASBVds and not to the phosphates.

Analogous spectral changes were obtained for Mg^2+^-binding to ASBVd(+), although to a smaller extent, about 50% less, than in the case of ASBVd(-).

It is worth noting that the conformation of magnesium-bound viroid at 20°C is not an active one, since the *in vitro* activity experiments show no self-cleavage kinetics.

### Mg^2+^-induced self-cleavage

**Figure 6:**
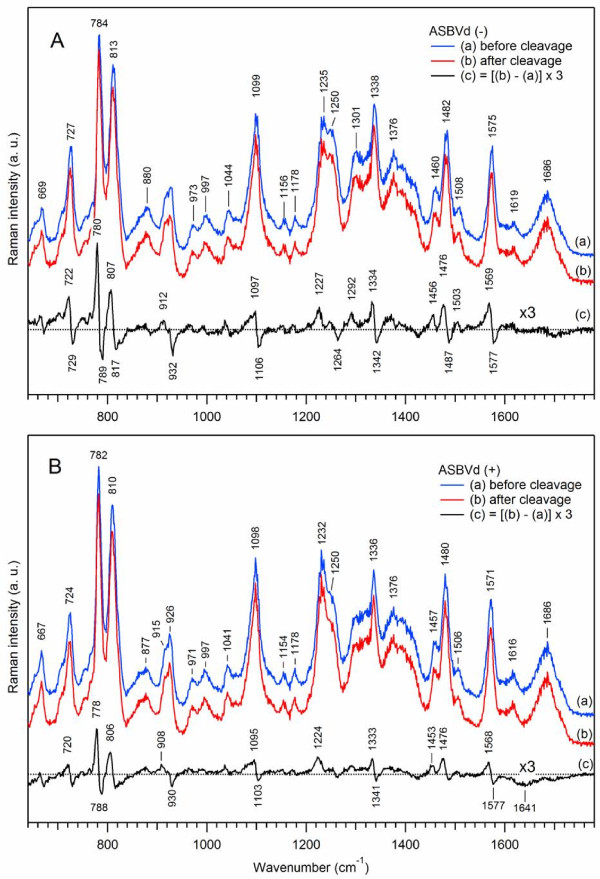
**Effect of Mg**^**2+**^**-induced self-cleavage on Raman spectra of ASBVds.** The Raman spectra of the minus (panel **A**) and plus (panel **B**) strands of ASBVd are obtained in cacodylate buffer, in the presence of 20 mM Mg^2+^. Blue curve (a) correspond to Raman spectrum taken before cleavage; red curve (b) correspond to Raman spectrum taken after cleavage (see text for details). All spectra were normalized using the Raman band at 1100 cm^-1^; the contributions from the cacodylate buffer and quartz cell were subtracted. The difference spectrum (c) (c = b-a, black curve) was multiplied by a factor of 3 to visually enhance the resulting spectral changes corresponding to Mg^2+^ induced self-cleavage.

Figure 6 presents Raman spectra of auto-catalytic self-cleaved minus (panel A) and plus (panel B) viroid strands. The experiment was performed as follows. The “before cleavage” sample (curves (a)) was the freshly Mg^2+^-bound viroids at 20°C. The “after cleavage” sample (curves (b)) was prepared by incubating the initial native viroid in the presence of 20 mM Mg^2+^ at 45°C for 4 hours and then bringing it back to 20°C, prior to the Raman experiment, so that both spectra (a) and (b) were recorded at 20°C. The difference Raman spectra (curves (c)) were obtained by 1:1 subtraction of the “before cleavage” sample from the “after cleavage” sample.

The immediate major result deduced from the analysis of the Raman difference spectra, for both viroid strands, is that the event of the viroid self-cleavage is not followed by strong structural perturbations (weak Raman intensity changes) and hence does not significantly alter the initial viroid structure. Nevertheless, we have observed minor but reproducible Raman band shifts upon Mg^2+^-induced self-cleavage in the spectrum of Figure 6 as compared to that obtained for Mg^2+^ binding without cleavage (Figure 5). Frequency downshifts of about 10 cm^-1^ take place with small intensity changes for the pyrimidine peak at ~784 cm^-1^ (6% in ASBVd(-) and 3% in ASBVd(+)), and about 4% intensity changes for the symmetric phosphodiester stretching vibration at 813 cm^-1^. The PO_2_^-^ band around 1100 cm^-1^ is also downshifted by ~9 cm^-1^ for both viroid strands, with similar small intensity changes of about 8%. Such frequency downshifts for the “after-cleavage” species are indicative of vibrational energy decrease of the phosphate groups. It is presumably a conformational consequence of self-cleavage (C-U and C-G) sites leading to the change from a 5′,3′ phosphate diester to a 2′,3′cyclic phosphate diester. Note that similar changes have been reported in Raman spectra of a model AMP cyclic compound [44,47]. In addition, the self-cleavage reaction perturbs both the frequency and intensity of the A stretching mode at 727 cm^-1^, as well as those of the sugar stretching mode around 929 cm^-1^. Furthermore, no difference in the r_stack_ parameter was observed. All these observations indicate that Raman spectroscopy is sensitive enough to reveal local conformational and torsional changes of the phosphate backbone (-C-O-P-O–C-), sugar puckering and base pairing induced by the cleavage of a single adjacent pair of (C,U) or (C,G) bases.

Finally, viroids of minus and plus strands exhibit very similar frequency shifts (Figure 6A(c) versus Figure 6B(c)), but differ in the amplitudes of spectral changes, being ~50% less pronounced for ASBVd(+) than for ASBVd(-). Our result is compatible with the *in vitro* activity measurements which indicate that about 60% and 10% of the RNA is cleaved in ASBVd(-) and ASBVd(+) strands respectively. Thus we conclude that structural perturbations due to Mg^2+^-induced self-cleavage are weaker for ASBVd(+), presumably because its structure is more rigid than that of ASBVd(-) and/or because ASBVd(+) is less active, leading to the reduced amount of the cleaved species.

## Conclusions

The results of the present study emphasize the power of the combined use of Raman structural markers, deuteration and temperature perturbation methods to analyze in detail the Raman spectra of the ASBVd minus and plus strands. The specific Raman markers provide not only qualitative but also quantitative information revealing the dynamic picture of the viroid structure.

Many structural details are obtained revealing that the backbone of ASBVd exhibits the A-type conformation with high double helical content, C3′-endo/anti sugar puckers and strong base-stacking. Nevertheless small specific differences are detected between the minus and plus viroid strands. The phosphodiester groups in ASBVd(+) are more rigid than in ASBVd(-), with slight differences in base-base interactions and ring vibrational coupling between purine bases and the riboses leading to local differences in the sugar puckering conformation. ASBVd(+) is shown to self-cleave 3.5 times less actively than ASBVd(-) (Figure 2).

The D_2_O perturbation experiments show that the changes in the ASBVd(-) and (+) spectra are more pronounced than in the case of the self-cleavage reaction. Another A-type conformation of the RNA is observed where r_conf_ decreases from 1.2 in H_2_O to 0.9 in D_2_O and where the frequencies of the doublet (785 cm^-1^ and 813 cm^-1^) downshift respectively to 11 cm^-1^ and 5 cm^-1^. In addition the increase of base-stacking ratio (r_stack_) clearly demonstrates that deuteration leads to a conformational change of the ribose-phosphate backbone and that the RNA structure is more rigid in ASBVd(+) than in ASBVd(-). Raman difference spectroscopy is shown to be a very sensitive technique to reveal small conformational perturbations upon solvent replacement, in the internal loops as well as in the hairpins of RNA dynamic structures.

Unfolding temperature at 65°C mainly perturbs the phosphodiester conformations as seen in the changes of the 785/813 cm^-1^ doublet and of the phosphodioxy band at 1100 cm^-1^. The perturbed spectra indicate mainly a disruption of the A-type structure of ASBVd through the double helical to random coil conformation transitions and the loss of stacking between bases (about 21%). No change in the sugar puckers but strong hydration and bond formation affecting the phosphodioxy charges are revealed from the downshift of phosphodioxy symmetric stretching at 1100 cm^-1^. The ASBVd(+) shows the same spectra perturbations but to a lesser extent and it is demonstrated that the positive strand is more stable than the negative strand.

Binding of Mg^2+^ to both viroid strands leads to small increases of the Raman intensities in the phosphodiester backbone modes with no changes in base-stacking and a moderate increase in H-bonding. The small increase in H-bonding is not due to increase in ionic strength of the magnesium cations, but rather due to magnesium effect on the specific bases of ASBVds (see Methods, Viroid preparation). The spectral changes are weaker for ASBVd(+) as compared to ASBVd(-). Magnesium binding to ASBVds at room temperature does not give rise to an active conformation. The active species is produced usually at a temperature of 45°C.

Self-cleavage of the RNA in ASBVd viroids is not followed by strong structural perturbations and hence does not significantly alter the initial viroid structure. However we were able to detect minor but readily measurable spectral changes that are different from that of simple Mg^2+^ binding. Viroid self-cleavage leads to very small perturbations in Raman intensities, but rather noticeable frequency downshifts of some Raman markers. The N-type sugar puckers decrease by ~5%. Such types of changes suggest that a local cleavage in a single adjacent pair of (C,U) bases could induce local conformational and torsional changes of the phosphate (-C-O-P-O-C-) backbone. No change in double helical content was detected. The spectral changes were weaker in the case of ASBVd(+), presumably because the structure of the positive strand is more rigid than that of the negative strand.

Finally spectral changes in Raman difference spectra due to external perturbations and self-cleavage could be plausibly associated with various structural changes in double helices, internal loops, hairpins and three-way junction in the two ASBVd structures as predicted in Ref. [11].

## Methods

### Plasmid construct

Plasmid *pBm*ASBVd was constructed by cloning the monomer ASBVd sequence into the PKS plasmid, restricted by the *Eco*R1 and *Bam*H1 enzymes, and the monomer sequence was extracted from the plasmid pBdASBVd containing a dimeric viroid cDNA [45].

### Viroid preparation

ASBVd (247 nt RNA single chains plus two guanylic residues added at the 5′ end for efficient transcription) were prepared by *in vitro* transcription. DNA templates used for transcription to synthesize the (-) and (+) ASBVd, were PCR amplified products obtained using respectively the oligonucleotides sense 5′-*TAATACGACTCACTATA*GGAAGAGATTGAAGACGAGTG-3′ containing the *T7 promoter* (italic font), and antisense 5′-GATCACTTCGTCTCTTCAGG-3′, or the oligonucleotides sense 5′-AAGAGATTGAAGAC GAGTG-3′ and antisense 5′ *TAATACGACTCACTATA*GGGATCACTTCGTCTCT TCAGG-3′ containing the *T7 promoter* (italic font) as primers in the presence of the *pBm*ASBVd plasmid. The recovered cDNAs were precipitated with ethanol, and the resulting pellets were dissolved in water. The transcription reactions performed by the T7 RNA polymerase were carried out overnight at 37°C in a final volume of 5 ml containing transcription buffer, each rNTP and RNase inhibitor (Fermentas). The transcriptions were stopped by treatment with RNase-free DNase I (Fermentas), for 30 min at 37°C, to degrade the DNA templates. The RNAs were precipitated with ethanol and resuspended in water, 0.05% xylene cyanol, and 50% deionized formamide. The resulting mixture was denatured for 2 min at 65°C prior to fractionation by denaturing (7 M urea), 10% polyacrylamide gel electrophoresis (PAGE, 19:1 ratio of acrylamide/bisacrylamide) using TBE buffer. Transcripts were detected by UV shadowing and the bands corresponding to full-length viroids of both polarities were excised, the RNA eluted overnight in 300 mM sodium acetate, pH 5.2, filtered through 0.22 μM filters, ethanol precipitated and dried. After dissolving in ultrapure water, the RNA concentrations were determined by UV absorbance (NanoVue GE Healthcare) and the samples were stored at -20°C.

For Raman experiments, 800 μg of dry RNA were suspended in 10 μl of water and denatured at 95°C for 45 sec and then slowly (3°C/min) renatured by cooling to 20°C. Salts and buffer were adjusted to the experimental conditions in a total volume of 10–20 μL. The final viroid concentration in the sample cell varied between 0.63 and 1 mM. The standard aqueous cacodylate buffer used in Raman experiments (if not indicated otherwise) was the following: H_2_O, 20 mM sodium cacodylate pH 7.2, 150 mM KCl, without Mg^2+^, at 20°C. The total ionic strength of the buffer is I_buffer_ = 170 mM, while that of added 20 mM Mg^2+^ is I_Mg2+_ = 60 mM. The addition of 20 mM Mg^2+^ in the solution does not change the pH of the cacodylate buffer. The standard deuterated aqueous cacodylate buffer was the following: D_2_O, 20 mM sodium cacodylate pH 6.8, 150 mM KCl, no Mg^2+^, at 20°C. The sample cell was thermostated within ±1°C. For measurements at elevated temperatures, a thin layer of oil was gently poured on top of the sample solution, to prevent evaporation.

### Gel electrophoresis experiments

Kinetics of the viroid cleavage was followed at 45°C in 50 mM cacodylate buffer pH 7.2 in the presence of 150 mM KCl and 20 mM Mg^2+^. Aliquots were removed from the incubating viroid samples at various times and quenched in one volume of stop solution (7 M urea, 50 mM EDTA, pH 7.5 and 0.01% xylene cyanol). Each aliquot was then loaded onto a denaturing gel (6% SDS-PAGE) to determine the fraction of the cleaved product. The total experiment time was ~3 hours.

### Raman experiments

Raman spectra of viroids were recorded using a home-built near-infrared (NIR) Raman setup [48]. Briefly, the excitation light at 780 nm was provided by continuous-wave Ti:Sapphire laser (Spectra Physics, model 3900S) pumped by an argon-ion laser (Spectra Physics Stabilite 2017). Laser light was focused into the specifically designed quartz sample cell (external dimensions 10×10×40 mm, internal dimensions 2×2×35 mm) filled with viroid solution (10–20 μL) by an infinity-corrected long working distance objective (Olympus MA10, M = 10X, NA = 0.25). Excitation power was attenuated to ~300 mW in the sample cell. The same objective was used to collect the Raman signal in a backscattering geometry and to deliver it onto the spectrograph (Acton SpectraPro 2500i) coupled with deep-depletion back-illuminated NIR CCD (Princeton Instruments SPEC-10 400BR/LN). The Raman light was focused on the spectrograph entrance (slit width 30 μm) by an achromatic lens with f = 75 mm. The Raman signal was separated from the laser light by two Semrock RazorEdge long pass filters (grade U). One filter, with λ_laser_ = 780 nm, was placed perpendicularly to the optical beam just before the focusing lens; another one, with λ_laser_ = 830 nm, was used as a dichroic beamsplitter at an angle of incidence of 45°: it reflected laser light at 780 nm and transmitted all the wavelengths longer than 785 nm. Raman spectra were acquired with the WinSpec software; further data treatment was performed using Igor Pro for Windows software. To obtain the viroid Raman spectrum, the contributions from the PBS and quartz cell were subtracted from the recorded spectrum, normalized on the water bending band around 1640 cm^-1^. Spectral resolution of Raman experiments was ~5 cm^-1^; frequency calibration was performed using Raman lines of toluene with absolute accuracy ±2 cm^-1^ and relative frequency position accuracy better than ±1 cm^-1^. The total accumulation time for one Raman spectrum was 10 to 30 min depending on the sample studied.

## Competing interests

The authors declare that they have no competing interests.

## Authors’ contributions

GHBH, SGK and MCM planned, conceptualized the study and wrote the manuscript. HK and JV purified the viroids and performed the in vitro activity measurements. HK and SGK ran Raman experiments. SGK and GHBH performed Raman data treatment and analysis. All authors read and approved the final manuscript.
